# Molecular Distance to Health Transcriptional Score and Disease Severity in Children Hospitalized With Community-Acquired Pneumonia

**DOI:** 10.3389/fcimb.2018.00382

**Published:** 2018-10-30

**Authors:** Rebecca G. Wallihan, Nicolás M. Suárez, Daniel M. Cohen, Mario Marcon, Melissa Moore-Clingenpeel, Asuncion Mejias, Octavio Ramilo

**Affiliations:** ^1^Division of Infectious Diseases, Nationwide Children's Hospital, Columbus, OH, United States; ^2^Center for Vaccines and Immunity, The Research Institute at Nationwide Children's Hospital, Columbus, OH, United States; ^3^Division of Emergency Medicine, Nationwide Children's Hospital, Columbus, OH, United States; ^4^Department of Pediatrics, The Ohio State University College of Medicine, Columbus, OH, United States; ^5^Biostatistics Core, The Research Institute at Nationwide Children's Hospital, Columbus, OH, United States

**Keywords:** pediatric pneumonia, transcriptional profile analysis, gene expression profiling, biomarker, community-acquired pneumonia

## Abstract

**Background:** Community-acquired pneumonia (CAP) is a leading cause of hospitalization and mortality in children. Diagnosis remains challenging and there are no reliable tools to objectively risk stratify patients or predict clinical outcomes. Molecular distance to health (MDTH) is a genomic score that measures the global perturbation of the transcriptional profile and may help classify patients by disease severity. We evaluated the value of MDTH to assess disease severity in children hospitalized with CAP.

**Methods:** Children hospitalized with CAP and matched healthy controls were enrolled in a prospective observational study. Blood samples were obtained for transcriptome analyses within 24 h of hospitalization. MDTH scores were calculated to assess disease severity and correlated with laboratory markers, such as white blood cell count, c-reactive protein (CRP), and procalcitonin (PCT), and clinical outcomes, including duration of fever and duration of hospitalization (LOS). Univariate and multivariable logistic regression were applied to assess factors associated with LOS and duration of fever after hospitalization.

**Results:** Among children hospitalized with CAP (*n* = 152), pyogenic bacteria (PB) were detected in 16 (11%), *Mycoplasma pneumoniae* was detected in 41 (28%), respiratory viruses (RV) alone were detected in 78 (51%), and no pathogen was detected in 17 (11%) children. Statistical group comparisons identified 6,726 genes differentially expressed in patients with CAP vs. healthy controls (*n* = 39). Children with confirmed PB had higher MDTH scores than those with RV (*p* < 0.05) or *M. pneumoniae* (*p* < 0.01) detected alone. CRP (*r* = 0.39, *p* < 0.0001), PCT (*r* = 0.39, *p* < 0.0001), and MDTHs (*r* = 0.24, *p* < 0.01) correlated with duration of fever, while only MDTHs correlated with LOS (*r* = 0.33, *p* < 0.0001). Unadjusted analyses showed that both higher CRP and MDTHs were associated with longer LOS (OR 1.04 [1–1.07] and 1.12 [1.04–1.20], respectively), however, only MDTH remained significant when adjusting for other covariates (aOR 1.11 [1.01–1.22]).

**Conclusions:** In children hospitalized with CAP MDTH score measured within 24 h of admission was independently associated with longer duration of hospitalization, regardless of the pathogen detected. This suggests that transcriptional biomarkers may represent a promising approach to assess disease severity in children with CAP.

## Background

In industrialized countries, community-acquired pneumonia (CAP) has an annual incidence of 36–40 per 1,000 children below the age of 5 years and 11–16 per 1,000 in children 5–14 years of age (Juvén et al., 2000). Although the mortality does not reach the levels reported in the developing world, there is significant morbidity and financial burden associated with pneumonia. In the United States it is second only to injuries as the most common reason for hospitalization in children <18 years of age (National Center for Health Statistics, 2011), with recent data reporting an overall incidence of 15.7 cases per 10,000 children and an incidence of 62.2 cases per 10,000 children under the age of two (Jain et al., 2015).

Despite its high incidence, the diagnosis and management of pediatric CAP remains a significant challenge to clinicians. First, a specific etiology is not identified in many cases (Wubbel et al., 1999; Juvén et al., 2000; Toikka et al., 2000; Moulin et al., 2001; Korppi, 2004; Michelow et al., 2004; Don et al., 2005), making targeted therapy difficult. Additionally, the clinical features of CAP are variable and may overlap with other respiratory diseases, such as asthma or bronchiolitis (Margolis and Gadomski, 1998; Lynch et al., 2004). This makes appropriate triage of children with CAP problematic in many cases, and there are currently no reliable tools to objectively classify patients according to disease severity, or to predict which patients will develop complications or need a higher level of care. In fact, guidelines for the diagnosis and management of CAP in children propose a number of areas for future research, including definition of risk factors for respiratory failure and hospitalization, and development of new diagnostic tests, not only to determine the etiology of CAP but to assess disease severity and response to therapy (Bradley et al., 2011).

The majority of studies that applied transcriptional profile analysis in children with infectious diseases have focused on the ability of this tool to aid in establishing the etiologic diagnosis of infections. A number of studies conducted in febrile children with bacterial and viral infections showed that transcriptional profiles can differentiate viral from bacterial infections with up to 90% accuracy (Ramilo et al., 2007; Herberg et al., 2016; Mahajan et al., 2016). Regardless, another potential application of transcriptional profile biomarkers in the clinical setting is the ability to help assess disease activity or disease severity in an objective manner (Chaussabel et al., 2008; Mejias et al., 2013). Molecular distance to health (MDTH) is a novel metric tool, which provides a single numeric score that summarizes the global perturbation of the transcriptional profile of each patient compared to healthy controls as the reference standard (Pankla et al., 2009). It has been shown to accurately classify the severity of the disease in patients with bacterial sepsis (Pankla et al., 2009), staphylococcal infections (Banchereau et al., 2012), as well as in children with respiratory viral infections (Mejias et al., 2013; Heinonen et al., 2016). Our hypothesis was that transcriptional profiling, specifically the MDTH score, would serve as an accurate biomarker of disease severity in children hospitalized with CAP.

## Materials and methods

### Patient population and healthy controls

This was an observational study involving a convenience sample of previously healthy children hospitalized with CAP at Nationwide Children's Hospital (NCH), Columbus, Ohio, between February 1, 2011, and May 10, 2012. Patients were reviewed for eligibility at the time of admission to the hospital. Inclusion criteria included age 2 months to 18 years, evidence of acute infection, signs or symptoms of respiratory illness, and radiologic confirmation of lower respiratory tract disease. Exclusion criteria included significant pre-existing medical conditions, use of immunomodulatory agents, prematurity <34 weeks in subjects younger than 2 years of age, and a primary diagnosis of bronchiolitis. Supplemental Table 1 includes all inclusion and exclusion criteria. Enrollment was completed and all samples were obtained within 24 h of admission. Healthy controls were enrolled during outpatient routine visits or minor elective surgical procedures not involving the respiratory tract. For the healthy control group, a clinical questionnaire was used, and those children with co-morbidities, use of systemic steroids, or presence of any illness within 2 weeks prior to enrollment were excluded. Written informed consent was obtained from parents/guardians before enrollment in accordance with the Declaration of Helsinki. The NCH Institutional Review Board (IRB) approved this study (NCH IRB 10-00028).

### Clinical data and microbiologic evaluation

Standard of care for children hospitalized with CAP at NCH during the study period included blood culture, complete blood count (CBC), C-reactive protein (CRP), nasopharyngeal (NP) swab for viral detection via direct fluorescent antibody (DFA), which included influenza A and B, parainfluenza 1, 2, and 3, adenovirus, RSV (Millipore; Billerica, MA, United States), and human metapneumovirus (HMPV; Diagnostic Hybrids; Athens, OH, United States), or polymerase chain reaction (PCR), and NP and/or oropharyngeal (OP) swab for *Mycoplasma pneumoniae* detection by PCR. If pleural fluid was obtained per standard care, samples were analyzed by routine bacterial culture and PCR assays for *Streptococcus pneumoniae, Streptococcus pyogenes*, and *M. pneumoniae*. Real-time PCR for *S. pneumoniae* was performed on a LightCycler® (Roche Diagnostics, Indianapolis, IN, United States) using a laboratory developed assay modified from Saukkoriipi et al. (2002) which targets a 278 bp segment of the pneumolysin *ply* gene (Marcon et al., 2009; Yu et al., 2011). *S. pyogenes and M. pneumoniae* PCRs were performed on an ABI 7500 Real-Time PCR System (Applied Biosystems, Carlsbad, CA, United States) using separate laboratory-developed assays. The *S. pyogenes* assay targeted an 86 bp segment of the pyrogenic exotoxin B (*speB*) gene (Marcon et al., 2004) and the *M. pneumoniae* assay targeted a 76 bp segment of the P1 adhesin protein (*p1ad*) gene (Hardegger et al., 2000).

In addition to samples obtained for routine clinical care, additional blood samples were obtained for measuring procalcitonin (PCT) concentration and for *S. pneumoniae* and *S. pyogenes* identification by PCR. Procalcitonin was measured using the VIDAS® platform (bioMerieux, Durham, NC). All blood PCRs and PCT assays were performed in batches after discharge and were therefore not available for clinical decision-making. In addition to testing NP specimens by DFA or PCR as per standard of care (as described above), NP specimens were also analyzed using both the xTAG® RVP and RVP *FAST* multiplex assays (Luminex, Austin, TX, United States) which included the detection of 13 viruses: respiratory syncytial virus (RSV) A and B, influenza A (non-specific A, H1, H3, and H5), influenza B, parainfluenza virus (PIV) 1–4, HMPV, rhinovirus/enterovirus (RV), adenovirus (ADV), coronavirus (NL63, 229E, OC43, HKU1, and SARS) and human bocavirus (HBV).

For the purposes of transcriptional profile analysis children were categorized into four groups according to detection of a viral or bacterial pathogen: (1) pyogenic bacteria, (2) *M. pneumoniae*, (3) respiratory viruses, or (4) undetermined. Patients were included in the pyogenic bacteria group if a bacterial pathogen was identified by culture or PCR from blood or pleural fluid, with or without a concomitant detection of a respiratory virus or *M. pneumoniae* in a NP specimen. Patients were included in the *M. pneumoniae* group by a positive PCR result from a NP or OP specimen, with or without a concomitant respiratory virus. Patients were included in the respiratory virus group by a positive result on any viral assay performed as standard of care or for research purposes, without detection of pyogenic bacteria or *M. pneumoniae*. Finally, patients were classified as undetermined if no pathogen was detected. Since current standard microbiologic diagnostic methods have limitations, especially for detection of bacterial pathogens, as blood cultures lack adequate sensitivity, we acknowledge that the group allocation based on pathogen detection is suboptimal. Nevertheless, the study was focused on identification of biomarkers to assess disease severity, instead of defining the pathogen-specific diagnostic biosignatures.

Electronic healthcare records were reviewed for demographic, clinical, laboratory, and radiographic data. Duration of fever after hospitalization (temperature ≥38°C), days of respiratory support (supplemental oxygen, non-invasive ventilation, and intubation), and duration of hospitalization (LOS) were used as clinical markers of disease severity. During the majority of the study period, there were no standard discharge criteria at our institution; the decision to discharge was made at the discretion of the attending physician.

### Transcriptional profile analysis and MDTH

Whole blood samples (1–3 mL) for microarray analyses from patients and age- and sex-matched healthy controls were collected in Tempus tubes (Applied Biosystems, CA, United States) and stored at −20°C until analyzed. Whole blood RNA was processed and hybridized into Illumina Human HT-12 v4 beadchips (47,323 probes) and scanned on the Illumina Beadstation 500 (Banchereau et al., 2012; Mejias et al., 2013; Heinonen et al., 2016). llumina GenomeStudio software was used to subtract background and scale average samples' signal intensity, and GeneSpring GX 7.3 (Agilent Technologies, Palo Alto, CA, United States) software to perform further normalization and analyses (Allantaz et al., 2007; Ramilo et al., 2007; Berry et al., 2010). Briefly, transcripts were first selected if they were present in at least 10% of all samples and had a minimum of two-fold expression change compared with the median intensity across all samples. Transcripts that passed this filter were then included in the quality control (QC) gene list used for downstream analyses. First, we conducted class comparisons (comparative analyses between predefined sample groups). For these analyses, each of the 3 pathogen-detection groups: (1) pyogenic bacteria, (2) *M. pneumoniae* (without respiratory viruses for the purpose of class comparisons), and (3) respiratory viruses was compared separately with the healthy control group, using Mann-Whitney (*p* < 0.01) with Benjamini-Hochberg multiple test correction and ≥1.25 fold change in expression level relative to the control group (Berry et al., 2010; Mejias et al., 2013). The goal of these initial analyses was to identify genes that were significantly differentially expressed in these three groups of patients with CAP compared with healthy controls, which will aid in the calculations of the MDTH scores. Next, we calculated the MDTH scores, a metric that converts the global transcriptional perturbation measured in each patient sample into a numeric value that can be incorporated into analyses of clinical variables. This analysis consists in comparing the expression values of all significantly differentially over and underexpressed genes and the fold difference of their expression values (MDTH score) from each individual pneumonia patient vs. the median values of healthy controls as a reference, as previously described (Pankla et al., 2009; Berry et al., 2010; Banchereau et al., 2012; Mejias et al., 2013). Data has been deposited in Gene Expression Omnibus (GEO) number GSE103119.

### Statistical analysis

For bivariate analyses, patient demographics' and clinical characteristics were compared using the chi-square or Fisher's exact test, as appropriate. Normally distributed continuous variables were compared using *t*-test or one way ANOVA and results expressed as means and standard deviation (SD). Non-normally distributed continuous variables were compared using the Mann–Whitney or Kruskal-Wallis tests and results expressed as medians and 25–75% interquartile range (IQR). Univariate and multivariable logistic regression were used to assess factors associated with three major outcomes of care: duration of fever, duration of respiratory support and total duration of stay (LOS). To allow for a better clinical interpretation, these outcomes were dichotomized by the median: (LOS by ≤2 and >2 days; respiratory support and duration of fever after hospitalization by ≤1 and >1 day). Covariates with *p* < 0.15 by univariate analysis were further included in multivariable models. Analyses were conducted using SAS 9.4 (SAS Institute, Cary, NC, United States).

## Results

### Demographics and pathogen detection

**Figure 1:**
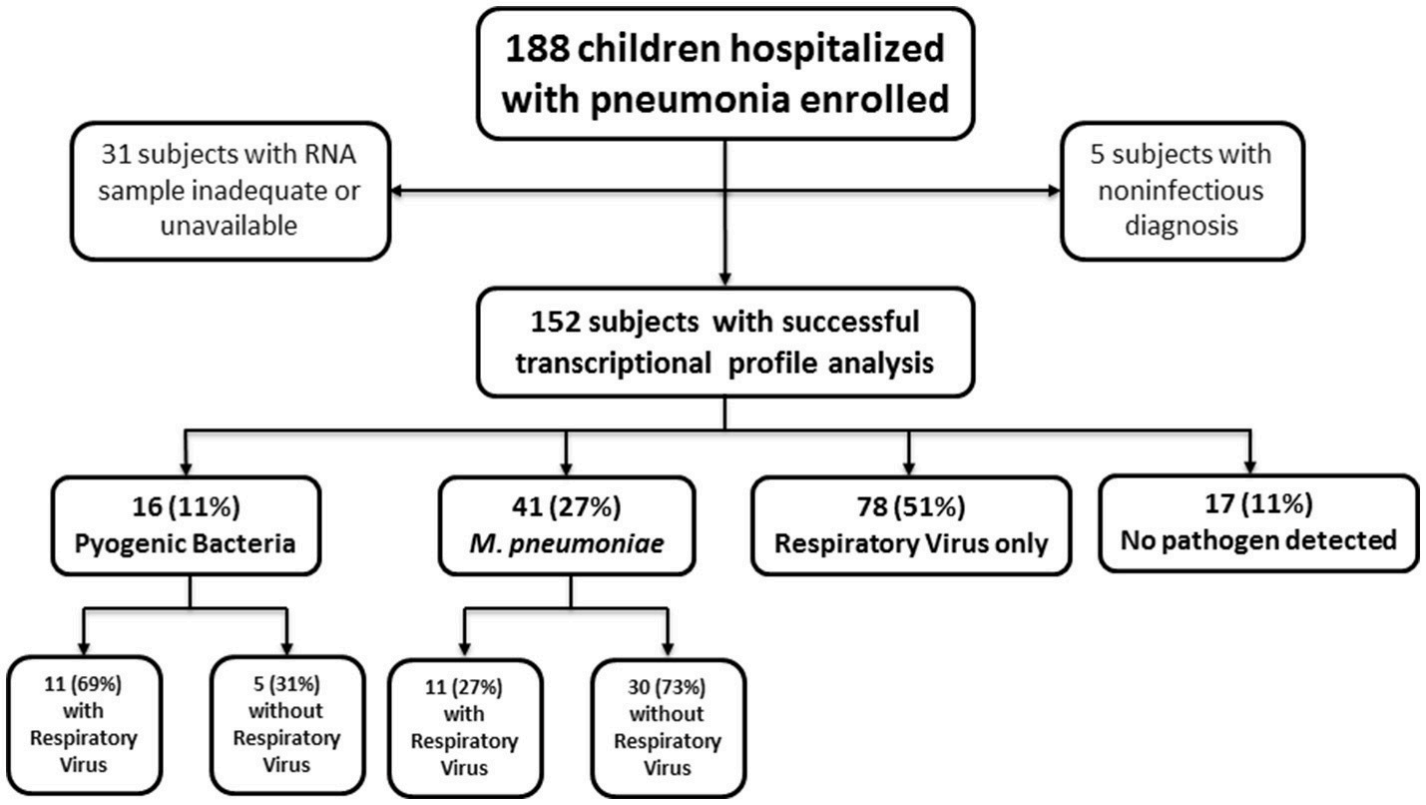
Flow chart depicting study population. Consort diagram of enrollment, exclusions, and final cohort. Subjects with noninfectious diagnoses and those with an inadequate or unavailable RNA sample were excluded from all analyses. One child with concomitant detection of *S. pneumoniae, M. pneumoniae*, and parainfluenza virus was categorized in the Pyogenic Bacteria group for all analyses.

One hundred and eighty-eight previously healthy children hospitalized with CAP were enrolled between February 1, 2011, and May 15, 2012. Five children were later excluded due to non-infectious diagnoses. Of the remaining 183 patients, 152 (83%) whole blood samples were available and successfully underwent transcriptional profile analysis. These patients were matched with 39 healthy controls for age, sex and race, that were also enrolled as part of the study. Pyogenic bacteria, with or without respiratory viruses, were detected in 16 (11%) children, including one child with concomitant detection of *S. pneumoniae, M. pneumoniae*, and parainfluenza virus. *M. pneumoniae*, with or without respiratory viruses, was detected in 42 (28%) children. Respiratory viruses alone were detected in 78 (51%) children. Seventeen (11%) children had no pathogen detected. Figure 1 summarizes enrollment and pathogens detected. Demographic, laboratory, and clinical data of patients and healthy controls are summarized in Table 1. Children in the *M. pneumoniae* group were older than children in the respiratory virus group (*p* < 0.001), but there were no other significant differences with respect to age, gender, or race among groups. A total of 9 (6%) children were admitted to the PICU; 1 (<1%) required mechanical ventilation, and an additional 4 (3%) required non-invasive ventilation.

**Table 1:** Demographic, laboratory, and clinical data of 152 patients and 39 healthy controls.

	**Healthy controls**	**All patients**	**Pyogenic bacteria^a^**	***M. pneumoniae*^b^**	**Respiratory viruses^c^**	**Undetermined**
	**(*n* = 39)**	**(*n* = 152)**	**(*n* = 16)**	**(*n* = 41)**	**(*n* = 78)**	**(*n* = 17)**
Age (years)^d, f^	3.6 [1.1–10.9]	5.1 [2.1–8.0]	5.8 [2.8–7.4]	8.0 [4.6–11.5]	3.6 [1.5–6.3]	6.5 [2.2–13.6]
Gender (M:F)	25:14	75:77	10:6	20:21	37:41	8:9
**RACE/ETHNICITY**						
White	30	96	10	26	45	15
Black	4	27	3	11	11	2
Hispanic	0	8	0	1	7	0
Other	5	21	3	3	15	0
WBC (Kcells/mm^3^)^d, e, g^	N/A	11.9 [8.9–17.4]	13.8 [10.2–23.4]	10.4 [7.3–15.1]	12.2 [10–17.6]	11.4 [7.1–20.5]
CRP (mg/dL)^d, e, g^	N/A	4.6 [2.1–17.4]	21.9 [6.8–27.2]	3.5 [1.8–4.8]	5 [2–8.6]	3.9 [2.1–14.5]
PCT (ng/mL)^d, e, g^	N/A	0.37 [0.13–2.3]	8.4 [4.1–26.6]	0.15 [0.06–0.48]	0.4 [0.2–2.4]	0.34 [0.05–0.57]
MDTH^d, h^	60 [31–156]	1747 [706–3800]	5712 [2556–8427]	1085 [657–2239]	1802 [576–3888]	1950 [816–2708]
Days of fever during hospitalization^d^	N/A	1 [0–2]	3 [2–5]	1 [0-2]	1 [0–2]	1 [0–1.5]
Days of respiratory support^d^	N/A	1 [0–2]	3 [1–4.8]	0 [0–2]	1 [0–2]	0 [0–1]
LOS (days)^d^	N/A	2 [1.3–3]	5.0 [3.8–8.8]	1.8 [1.1–2.4]	2.0 [1.2–2.7]	2.2 [1.6–3.3]

a Pyogenic bacteria (PB) group includes 5 children with only PB, 10 children with PB+ Respiratory virus, and 1 child with PB+Respiratory virus+M. pneumoniae; blood PCR for S. pneumoniae and S. pyogenes was performed in 110 patients.

b M. pneumoniae group includes 30 children with Mycoplasma only and 11 children with M. pneumoniae +virus; M. pneumoniae PCR was performed in 138 patients.

c Respiratory virus testing was performed in 146 patients.

d Median [IQR].

e WBC, CRP, and PCT values were available for 147, 139, and 122 patients, respectively.

f Children in the M. pneumoniae group were older than children in the respiratory virus group (p < 0.001); there were no other significant differences with respect to age, gender, or race among groups.

g Children with detection of pyogenic bacteria had higher MDTH, CRP, and PCT values than those with detection of respiratory viruses (p < 0.05, p < 0.01, and p < 0.001, respectively) or M. pneumoniae detected alone (p < 0.01, p < 0.001, and p < 0.0001, respectively), as well as higher CRP and PCT than children in the no pathogen detection group (p < 0.05 and p < 0.001, respectively).

h *MDTH scores were significantly higher in all pneumonia groups when compared with healthy controls (p < 0.0001)*.

**Figure 2:**
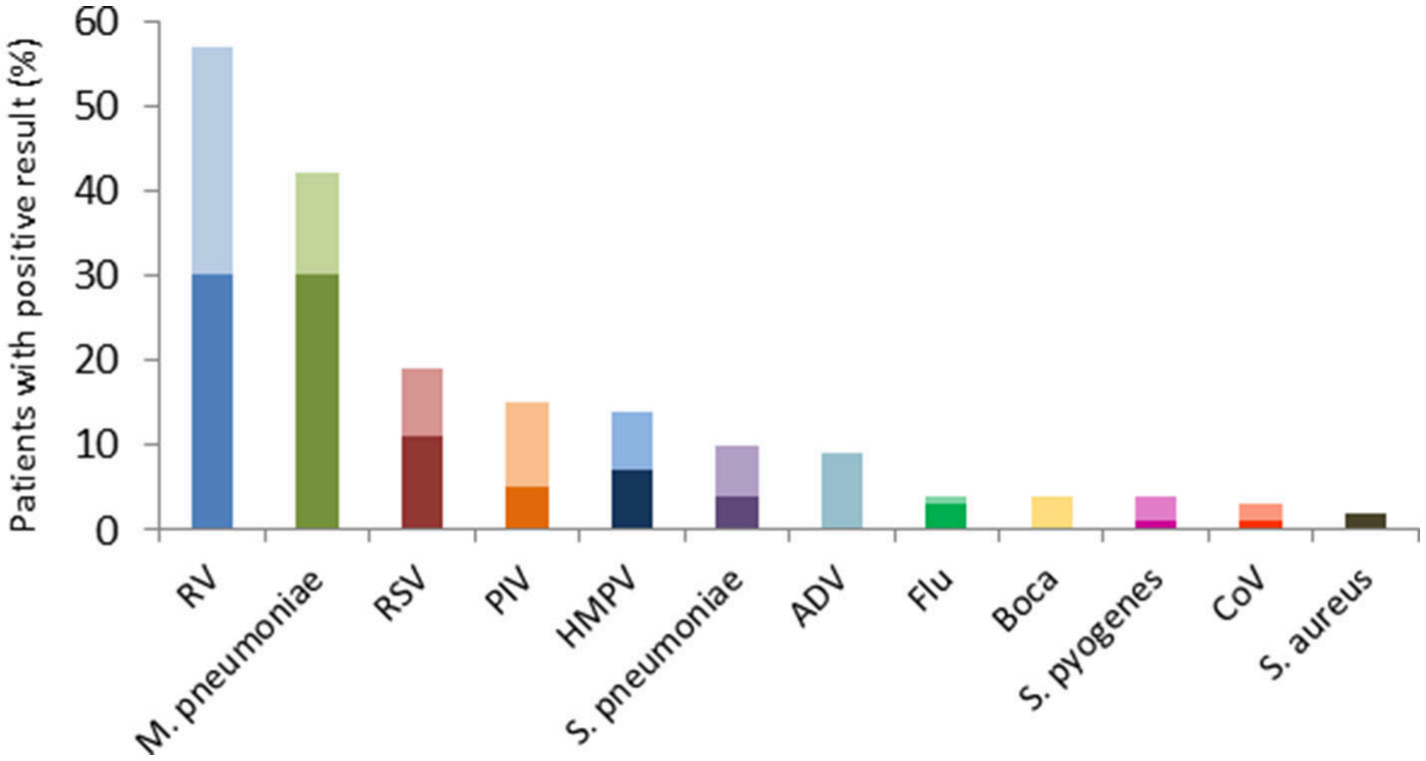
Pathogens detected in 152 children hospitalized with CAP. Pyogenic bacteria were detected in 16 (11%), M. pneumoniae was detected in 42 (28%), and 125 respiratory viruses were detected in 98 (64%) children. The dark bars represent detection of single pathogen, and the lighter bars represent co-detection with at least one other pathogen. Adenovirus and bocavirus were only detected in combination with other pathogens, while *S. aureus* was detected as the sole pathogen in 2 patients. RV, Rhino/enterovirus; RSV, Respiratory syncytial virus; PIV, Parainfluenza virus; HMPV, Human metapneumovirus; ADV, Adenovirus; Flu, Influenza virus; Boca, Bocavirus; CoV, Coronavirus.

*Streptococcus pneumoniae* (*n* = 10, 62%) was the most frequent pyogenic bacterium detected, followed by *S. pyogenes* (*n* = 4, 25%), and *S. aureus* (*n* = 2, 13%). Of 147 children tested for respiratory viruses, 125 viruses were detected in 98 (67%) children. As shown in Figure 2, rhinovirus/enterovirus (*n* = 57, 46%) was the most frequent virus detected, followed by RSV (*n* = 19, 15%), parainfluenza virus (*n* = 15, 12%), and human metapneumovirus (*n* = 14, 11%). Of the 98 with any virus detected, multiple viruses were detected in 22 (22%) children.

### Transcriptional profile analysis and MDTH scores

**Figure 3:**
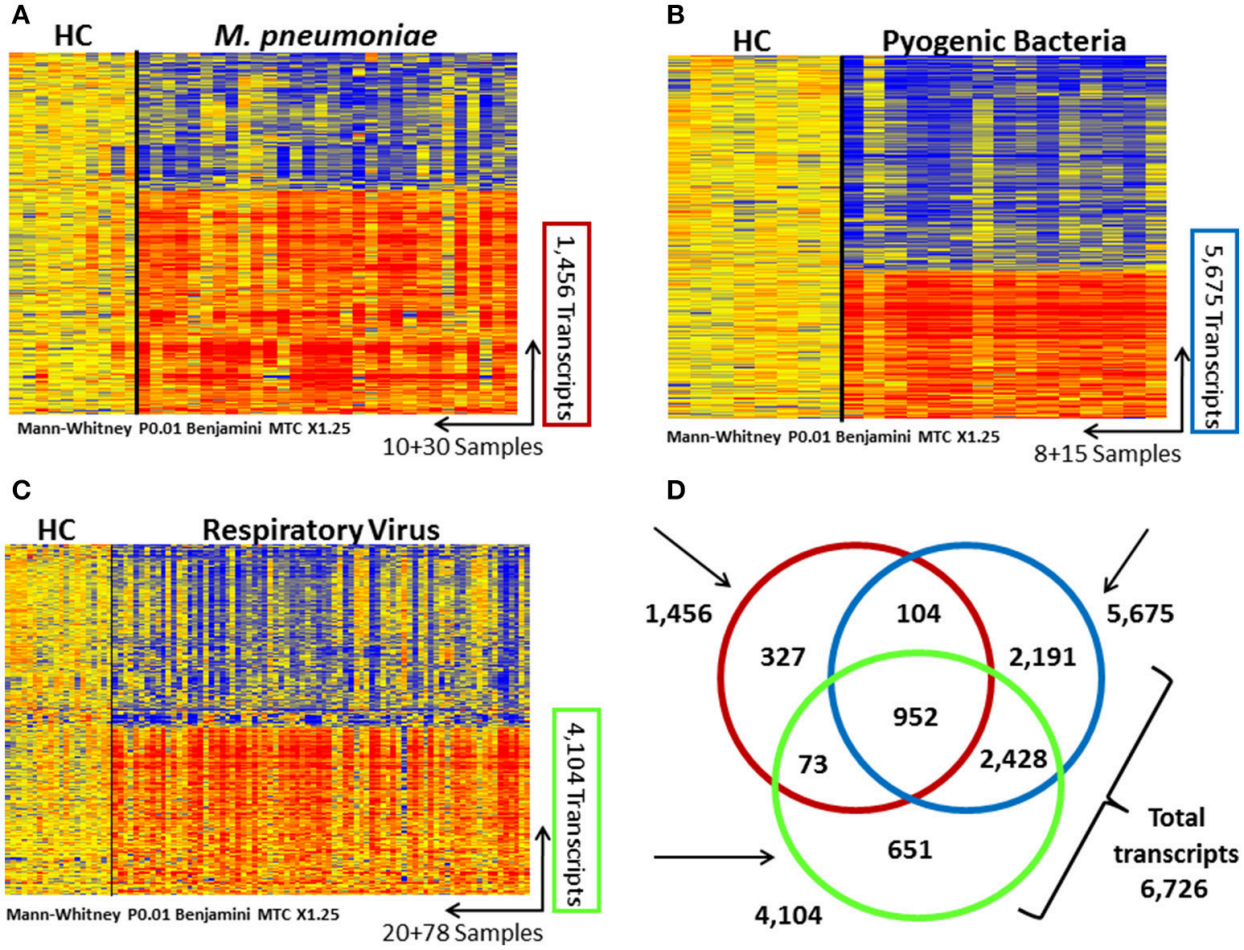
Transcriptional profiles in children with community-acquired pneumonia (CAP) caused by pyogenic bacteria, respiratory viruses, and *M. pneumoniae*. Transcripts are represented in rows, and individual subjects in columns. Normalized log ratio levels are indicated in red (overexpressed) or blue (underexpressed), as compared with the median expression of the healthy controls (HC). Heat-maps represent the transcriptional profiles of 10 healthy controls and 30 patients with *M. pneumoniae* CAP based on 1,456 transcripts **(A)**; 8 healthy controls and 15 patients with bacterial CAP based on 5,675 transcripts **(B)**, and 20 healthy controls and 78 patients with viral CAP based on 4,104 transcripts **(C)**. All transcripts were identified after applying a nonparametric test (Mann–Whitney) (*P* < 0.01), 1.25-fold change, and Benjamini–Hochberg multiple-test correction. **(D)** Venn diagram displaying the overlap among the significant transcripts identified in the 3 CAP groups. A total of 6,726 differentially expressed genes were identified in children with CAP due to pyogenic bacteria, respiratory viruses, and *M. pneumoniae*.

Statistical group comparisons identified 5,675 differentially expressed transcripts between children with pyogenic bacteria CAP and healthy controls, 1,456 transcripts between those with detection of *M. pneumoniae* and healthy controls, and 4,104 transcripts between children with detection of only respiratory viruses and healthy controls. The combination of these genes derived from pair-wise comparisons resulted in a total of 6,726 genes differentially expressed in children with CAP with detection of any of these pathogens (Figure 3). While 952 (15%) genes were shared among all pathogen groups, 2,191 (35%) were specific to pyogenic bacteria, 651 (10%) were specific to respiratory viruses, and 327 (5%) were specific to *M. pneumoniae*. This gene list of 6,726 genes was then used to calculate the MDTH scores. MDTH scores were significantly higher in all pneumonia groups when compared with healthy controls (*p* < 0.0001; Table 1). Children with detection of pyogenic bacteria had higher MDTH, CRP, and PCT values than those with detection of respiratory viruses (*p* < 0.05, *p* < 0.01, and *p* < 0.001, respectively) or *M. pneumoniae* detected alone (*p* < 0.01, *p* < 0.001, and *p* < 0.0001, respectively), as well as higher CRP and PCT than children in the no pathogen detection group (*p* < 0.05 and *p* < 0.001, respectively). Additionally, children with MDTH scores above the median value (MDTH >1,747) were more likely to be prescribed a course of antibiotics than those with MDTH scores at or below the median (95 vs. 78%; *p* = 0.004).

**Figure 4:**
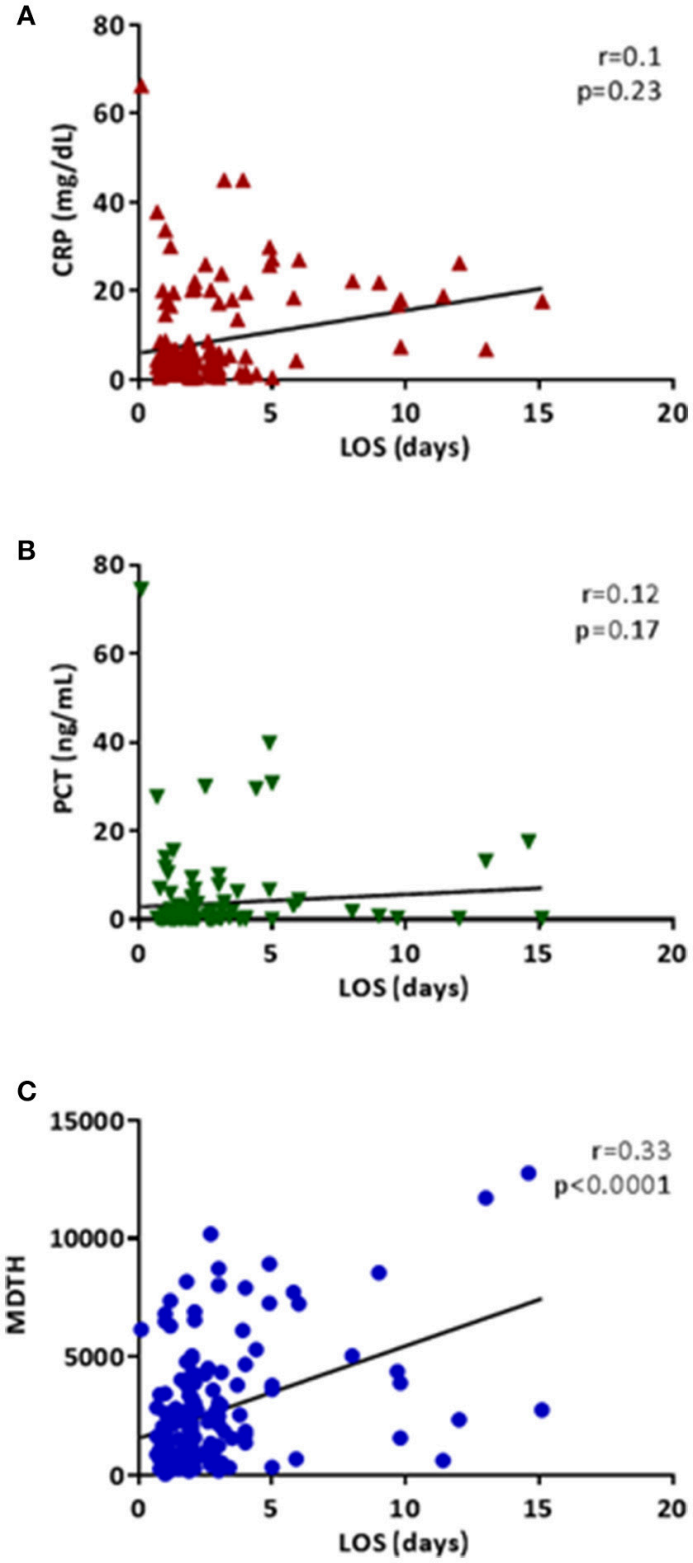
Correlations of inflammatory markers and MDTH with length of hospitalization (LOS). Each plot represents the correlation between length of hospitalization in days (x-axis) and CRP (**A**; red triangles), PCT (**B**; green inverted triangles), and MDTH (**C**; blue circles) on the y-axes. All correlations were performed using Spearman's correlation.

White blood cell counts at the time of admission to the hospital were not different among groups and did not correlate with any clinical marker of disease severity, including days of respiratory support, days of fever after hospitalization, or LOS. However, CRP (*r* = 0.39; *p* < 0.0001), PCT (*r* = 0.39; *p* < 0.0001), and MDTH (*r* = 0.24; *p* = 0.003), all measured on admission, correlated with days of fever after hospitalization (Supplemental Figure 1). In univariate analyses, only MDTH scores measured on admission correlated with LOS (*n* = 152; *r* = 0.33, *p* < 0.0001; Figure 4).

### Multivariable analysis

For this analysis we identified three clinical outcomes: days of respiratory support, duration of fever, and duration of hospital stay (LOS). For days of respiratory support, no variables of interest were significant in univariate analyses, so multivariable analysis was not performed. Table 2 shows the results for unadjusted and adjusted analyses for duration of fever after hospitalization, which was dichotomized in ≤1 and >1 day based on median duration of fever. By univariate analyses, higher MDTH was significantly associated with longer duration of fever. This association was not significant in the multivariable assessment. In regards to longer LOS (also dichotomized by the median in ≤2 and > 2days), univariate analyses showed that higher CRP and MDTH scores were both associated with increased LOS, but gender, race, age, WBC, and PCT were not. In the multivariable analyses, MDTH was the only biomarker significantly associated with longer LOS.

**Table 2:** Risk factors for duration of fever and length of hospitalization.

**Variable**	**Risk factors for fever**				**Risk factors for LOS**^**a**^			
	**Unadjusted OR^b^** **(95% CI)**	***p*-value**	**Adjusted OR** **(95% CI)**	***p*-value**	**Unadjusted OR** **(95% CI)**	***p*-value**	**Adjusted OR** **(95% CI)**	***p*-value**
Female	0.81 (0.41,1.57)	0.53	-	-	1.13 (0.59,2.16)	0.72	-	-
Race: Black vs. Other	1.25 (0.36,4.32)	0.78	-	-	2.2 (0.62,7.79)	0.59	-	-
Race: Hispanic vs. Other	0.83 (0.13,5.35)	0.64	-	-	1.92 (0.33,11.03)	0.91	-	-
Race: White vs. Other	1.5 (0.53,4.21)	0.33	-	-	2.49 (0.84,7.35)	0.26	-	-
Age (years)	1.01 (0.94,1.09)	0.73	-	-	1.06 (0.98,1.14)	0.14	1.1 (1.00,1.21)	**0.04**
WBC	1.04 (0.99,1.08)	0.56	1.00 (0.94,1.06)	0.86	1.03 (0.99,1.08)	0.12	1.02 (0.96,1.09)	0.45
CRP	1.06 (1.02,1.1)	0.69	0.98 (0.93,1.03)	0.37	1.04 (1,1.07)	**0.047**	1.00 (0.95,1.06)	0.95
PCT	1.04 (0.99,1.09)	0.09	0.99 (0.94,1.05)	0.84	1.02 (0.98,1.06)	0.40	0.99 (0.94,1.05)	0.85
MDTH, *per 500 point increase*	1.11 (1.03,1.19)	**0.0037**	0.95 (0.87,1.03)	0.22	1.12 (1.04,1.20)	**0.0016**	1.11 (1.01,1.22)	**0.03**

a LOS, Length of hospitalization.

b OR,odds ratio. Duration of fever and LOS were dichotomized by the cohort median values which were 1 day for fever and 2 days for duration of hospitalization. For every 500-point increase in MDTH scores there was a ~12% increased odds of LOS >2 days. *Bolded items indicate p < 0.05*.

## Discussion

Previous studies have shown the potential value of transcriptional profiling and the MDTH score for assessment of disease severity in a number of infections, in both adults and children. Most of those studies included patients infected by a single bacterial (i.e., melioidosis, *S. aureus*, tuberculosis) or viral (RSV, rhinovirus) pathogen (Pankla et al., 2009; Berry et al., 2010; Banchereau et al., 2012; Mejias et al., 2013). However, its application in the context of pediatric CAP caused by a variety of respiratory pathogens has not been evaluated previously. In the present study, we identified a transcriptional score (MDTH score), that significantly correlated with inflammatory markers, such as WBC, CRP, and PCT, and was independently associated with disease severity as defined by duration of hospitalization in children with CAP, regardless of the pathogen or pathogens detected using current microbiologic diagnostic assays.

Severity scoring systems have been validated for adults with lower respiratory tract infection (Neill et al., 1996; Fine et al., 1997; Lim et al., 2003; Capelastegui et al., 2006) but such tools are lacking for children evaluated in the developed world. Well-defined criteria for hospitalization of children with CAP have been recommended in guidelines (Bradley et al., 2011), but there is also a subjective component. A recent study from the United States showed promising results in using risk models, combining patient, laboratory, and radiographic characteristics, to predict severe pneumonia in children, but these have yet to be validated prospectively in larger cohorts (Williams et al., 2016). Multiple studies have suggested a limited role for inflammatory markers, such as peripheral WBC count, CRP, erythrocyte sedimentation rate, and PCT, in the diagnosis and management of pneumonia in children. It is difficult to define a cut-off for any of these values that is both sensitive and specific. In a study of 100 children with CAP, Don et al reported higher PCT levels in hospitalized patients compared to outpatients, but there was no further description of these values as related to disease severity (Don et al., 2007). More recently, lower PCT values were associated with a reduced risk of detection of pyogenic bacteria in a large cohort of children with pneumonia in the U.S. (Stockmann et al., 2017) Michelow et al examined levels of 15 cytokines in 55 children with CAP and found that only IL-6 correlated with indicators of disease severity (Michelow et al., 2007). Esposito and colleagues evaluated the role of a PCT-based algorithm to guide antibiotic therapy in children with mild/moderate CAP (Esposito et al., 2011) and showed that application of this algorithm was associated with reduced antibiotic use. It will be important to evaluate this PCT-based algorithm in children with severe disease. Accurate and rapid identification of children at higher risk of morbidity is needed to allow improved and more objective assessment of the need for hospitalization, faster initiation of appropriate antimicrobial therapy, and ultimately improved clinical outcomes.

Although the present study is the first to examine the value of the MDTH score in children with CAP, this tool has been applied to other patient populations with a variety of infections. In patients with pulmonary TB, MDTH scores correlated with both the extent of disease and clinical improvement after therapy (Berry et al., 2010). In patients with *S. aureus* infections, MDTH correlated with laboratory parameters such as CRP, WBC, and neutrophil counts and showed increased values with disease dissemination (Banchereau et al., 2012). MDTH scores were also evaluated in infants and children with respiratory infections caused by RSV and rhinovirus (Mejias et al., 2013; Heinonen et al., 2016; Jong et al., 2016). In children with RSV infection, the MDTH score at admission correlated with a clinical disease severity score, as well as with duration of supplemental oxygen and duration of hospitalization. Additionally, MDTH scores were decreased at follow-up visits, demonstrating its potential utility in monitoring response to therapy (Mejias et al., 2013). Application of the MDTH scores in children with detection of rhinovirus by PCR allowed discrimination between children with symptomatic respiratory infections vs. those with asymptomatic detection (Heinonen et al., 2016).

The present study has a number of strengths. First, it includes patients with a broad range of ages, increasing the generalizability of the results and highlighting the potential impact of the MDTH score in the pediatric population. Second, all patient samples were obtained within 24 h of hospitalization, reducing the potential influence of the time of sample collection. Finally, we did not limit our analysis to one specific pathogen or class of pathogens. While previous studies primarily focused on infection due to a single organism, we focused on a clinical syndrome with many etiologic agents. Thus, in addition to variability in clinical presentation and duration of illness prior to hospitalization, there was heterogeneity in the etiology of CAP. However, despite these factors, we were still able to show an association between the MDTH score and more traditional markers of inflammation, and most notably with the duration of hospitalization.

The study also has limitations. First, complete microbiologic data are lacking in some patients. Furthermore, the fact that current methods for bacterial detection are suboptimal, and although patients were classified according to the type of pathogen detected, we suspect that the pathogen groups may include misclassified patients. This is especially true in the group of patients with virus detected only, as this group may include patients with viral-bacterial coinfections. It should be mentioned, however, that the goal of the present study was not the diagnosis of specific pathogens or discrimination between bacterial and viral etiologies but rather the identification of a biomarker to objectively assess disease severity. Another potential limitation is that healthy controls were not comprehensively tested for respiratory pathogens, although children with current or history of respiratory symptoms within 2 weeks were excluded from the healthy control group. Additionally, analyses were not corrected for duration of illness prior to hospitalization, which may have caused variability in the MDTH scores within pathogen groups. Nevertheless, this could also be considered a strength of the study; because, despite the variability in duration of symptoms observed when managing children with CAP in clinical practice, we were able to identify an objective score to classify patients according to disease severity. Our cohort only included hospitalized patients in a single center in central Ohio, so future studies including ambulatory and hospitalized children from diverse geographic locations are warranted. Even at a single institution, discharge criteria were not standardized during the majority of the study period. Therefore, practice variability among individual clinicians may have influenced the duration of hospitalization. Finally, we did not obtain samples directly from the lower respiratory tract as only a minority of patients required invasive ventilator support and thus data from peripheral blood may not reflect immune response at the primary site of infection. Notwithstanding, using blood samples to measure the host response to respiratory pathogens and calculate the MDTH score will facilitate implementation in the clinical setting.

In summary, this initial study suggests that in children with CAP, MDTH scores may allow a more precise severity classification than current laboratory markers and routine application of this tool may enhance our clinical decision making process by providing an objective assessment of disease severity. Future studies are warranted which include larger numbers of children with CAP in both ambulatory and inpatient settings, patients with severe disease admitted to the PICU, and combining MDTH with other tools and/or markers to improve evaluation of disease severity.

## Data availability

Data has been deposited in Gene Expression Omnibus (GEO) number GSE103119.

## Author's note

Portions of this work were presented at the Pediatric Academic Societies Meeting, Washington, DC, May 4-7, 2013.

## Author contributions

RW, NS, DC, AM, MM, MM-C, and OR contributed to the conception and design of the study. RW, DC, AM, and OR recruited subjects. RW organized the database. RW and MM-C performed the statistical analyses. RW wrote the first draft of the manuscript. MM-C, NS, AM, and OR wrote sections of the manuscript. All authors contributed to manuscript revision, read and approved the submitted version.

### Conflict of interest statement

AM and OR have received research grants from Janssen. AM has received fees for participation in Advisory Boards from Janssen and lectures from Abbvie. OR has received fees for participation in Advisory Boards from Abbvie, Janssen and Regeneron, and for lectures from Abbvie, Pfizer, and Johnson and Johnson. The remaining authors declare that the research was conducted in the absence of any commercial or financial relationships that could be construed as a potential conflict of interest.

## Supplementary material

Supplemental Figure 1 Correlations of inflammatory markers and MDTH with duration of fever. Each plot represents the correlation between duration of fever in days (x-axis) and CRP (**A**; red triangles), PCT (**B**; green inverted triangles), and MDTH (**C**; blue circles) on the y-axes. All correlations were performed using Spearman's correlation.

Supplemental Table 1 Complete inclusion and exclusion criteria.
