# Supplementary figures and images for: Molecular Distance to Health Transcriptional Score and Disease Severity in Children Hospitalized With Community-Acquired Pneumonia
Source: Front Cell Infect Microbiol. 2018 Oct 30;8:382. doi: 10.3389/fcimb.2018.00382 (PMC6218690; doi:10.3389/fcimb.2018.00382)

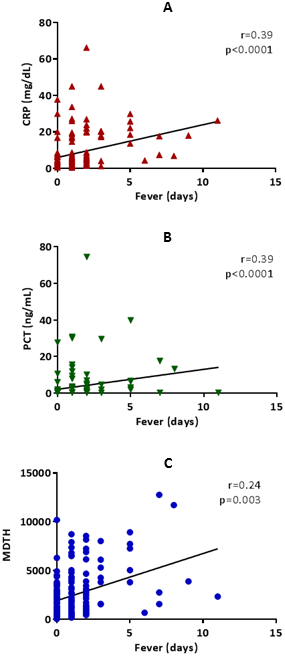

Supplement: Supplemental Figure 1 — Correlations of inflammatory markers and MDTH with duration of fever. Each plot represents the correlation between duration of fever in days (x-axis) and CRP (A; red triangles), PCT (B; green inverted triangles), and MDTH (C; blue circles) on the y-axes. All correlations were performed using Spearman's correlation. [file Image_1.TIF]
