# Supplementary material for: Molecular Distance to Health Transcriptional Score and Disease Severity in Children Hospitalized With Community-Acquired Pneumonia
Source: Front Cell Infect Microbiol. 2018 Oct 30;8:382. doi: 10.3389/fcimb.2018.00382 (PMC6218690; doi:10.3389/fcimb.2018.00382)
Supplement: Supplemental Table 1 — Complete inclusion and exclusion criteria. [file Data_Sheet_1.docx]

**Supplemental Table 1.** Complete inclusion and exclusion criteria.

| **Inclusion Criteria** | **Exclusion Criteria** |
| --- | --- |
| 1. **Hospitalized children ages 2 months-18 years** 2. **Evidence of an acute infection, including any one of the following:**    1. Fever or hypothermia: Documented temperature (rectal or oral) ≥38° C or <35.5° C    2. Reported fever, chills, or feeling feverish without documentation    3. Abnormal white blood cell count, either leukocytosis or leukopenia 3. Age <5 years: >15,000/cm^3^ or <5,500/cm^3^ 4. Age ≥5 years: >11,000/cm^3^ or <3,000/cm^3^ 5. **Signs or symptoms of respiratory illness, with at least one of the following symptoms:**    1. Documented tachypnea       1. Age 2 months to less than 12 months: > 50 breaths/minute       2. Age 12 months to 5 years: > 40 breaths/minute       3. Age greater than 5 years: > 25 breaths/minute    2. Cough    3. Abnormal breath sounds on auscultation (e.g., rales, rhonchi, wheezing, dullness)    4. Sputum production    5. Chest pain    6. Dyspnea or shortness of breath    7. Acute respiratory failure 6. **Radiologic confirmation of pneumonia (i.e. infiltrate on chest x-ray or chest CT)** | 1. **Patients with significant pre-existing medical conditions, including:** 2. Chronic lung disease (excluding asthma) 3. Cardiac disease 4. Neuromuscular disease 5. Proven or suspected immunodeficiency 6. Hematologic disorder or malignancy 7. **Patients receiving immunomodulatory agents, including but not limited to:** 8. Corticosteroid use for >2 weeks within the previous 6 weeks 9. Monoclonal antibodies 10. Tumor Necrosis Factor-alpha inhibitors 11. Cyclosporine 12. Azathioprine 13. **Prematurity <34 weeks if subject <2 years old** 14. **Primary diagnosis of bronchiolitis** |
